# Hair for brain trade-off, a metabolic bypass for encephalization

**DOI:** 10.1186/2193-1801-3-562

**Published:** 2014-09-27

**Authors:** Yosef Dror, Michael Hopp

**Affiliations:** Biochemistry and Human Nutrition, Faculty of Agriculture, The Hebrew University, 76100 Rehovot, Israel; Department of Geography, Tel Aviv University, Tel Aviv, Israel

**Keywords:** Hair, Brain, Methylation, Encephalization, Keratin composition, Sialic acid hydroxylation

## Abstract

Hair loss in humans is perplexing and raises many hypothetical explanations. This paper suggests that hair loss in humans is metabolically related to encephalization; and that hair covered hominids would have been unable to evolve large brains because of a dietary restriction of several amino acids which are essential for hair and brain development. We use simulations to imply that hair loss must have preceded increase in brain size & volume. In this respect we see hair loss as a major force in human evolution. We assume that hair reduction required favorable climatic conditions and must have been quick. Using evolutionary and ecological time scales, we pinpoint hair loss to a period around 2.2-2.4 million years ago. The dating is further supported by a rapid selection at that time of the sialic acid deletion mutation which may have protected growing human brains against calcium ion flux. In summary we view encephalization, in part, as a metabolic trade-off between hair and brain. Other biochemical changes may have intervened in the process too; and the deletion mutation of sialic acid hydroxylation may have been involved as well.

## Background

Hairlessness distinguishes humans from most mammals and all other primates. Along with bipedalism and large brains it is the most visually distinctive human characteristic. However, while brains and locomotion have clear functional and evolutionary advantages, the utility of hairlessness is less evident and it is often viewed as a mere curiosity. Since hair cannot be traced in the fossil record, explanations on its evolutionary loss are largely based on conjecture and have given rise to many intuitive non-verifiable explanations.

In this paper we propose that the evolution of human hairlessness is directly tied to brain growth and therefore merits close investigation. Our theory is based on biochemical and metabolic principles for which there is sufficient evidence to allow us to estimate that the human brain began to grow only after most hair cover was lost. Combining metabolism with cellular biochemistry we are further able to postulate that hair was lost and brains began to grow some 2.2-2.4 (million years ago).

Our proposed model does not upset the accepted view of human development. Rather, it focuses attention on a critical period in human evolution and provides biochemical and molecular insights to explain the unique emergence of a hairless large brained bipedal hominid at the end of this period. We focus on the primary utility of hair loss in humans and avoid putative secondary advantages of hairlessness like evaporative cooling (Carrier 1984) or brain heat stress (Fiailkowski 1986); and we sidestep hair loss in other species such as the case in cetaceans (Chen et al. 2013).

Briefly stated, all hair contains large amounts of several amino acids that are essential for brain development and function, but are in limited quantity in food. Our data show that hair and brains are in direct competition over this limited resource to such extent that a furry hominid would not have been able to develop a large brain. Therefore, we suggest that hair loss in humans must have preceded brain growth.

Because of the need for amino acids, our data further implies that encephalization could only develop in an omnivorous species.

### Protein, hair and brains

Like all tissue, hair and brain, are made up of protein which, in turn, is made up of amino acids building blocks. Some amino acids are synthesized internally and the rest are obtained from food. Amino acids which must be derived from external sources are called ‘essential’. If they are not supplied in sufficient quantity they can form developmental bottlenecks. Our argument is based on four essential amino acids, methionine, cysteine, arginine and tyrosine. These amino acids and their derivatives are in limited supply in food, are vital for brain development and function, and are also structural components of all hair.

Hair and brain are in a direct conflict over the supply of these scarce amino acids. This conflict is based on the nature of hair and on distinctive metabolic requirements of the brain, including methylation, antioxidant activity and osmolite capacity. Mammalian bodies can normally divert amino acids from one tissue to another to address temporary needs, but hair is a dead structure and amino acids deposited in it cannot be redirected for other need. Under conditions of limited supply, the deposition of protein in hair represents an amino acid loss which is tolerable in a small brained mammals or in a hairless human, but would be a limiting factor to brain growth and function in a hair covered hominid.

From an evolutionary perspective, this hair-brain trade-off would have conferred a metabolic advantage on hominids with less hair at a time when rapid brain development was a primary selective factor. Hair loss may have preceded encephalization, or could have been accelerate by it. This scenario locates human hair loss at a critical crossroads of human development and helps pinpoint it on the evolutionary timeline.

To develop our case we first briefly summarize the general role of amino acids and review the metabolic amino acid requirements of the brain. We next establish the interdependence of encephalization and hair loss by means of a metabolic estimation based on proto human and on primate nutrition. Finally we propose an evolutionary scenario to account for brain growth and hair loss.

### Amino acid requirements

Proteins are made up of 20 principal amino acid building blocks. Plants, bacteria and *archeae* synthesize all amino acids from other organic compounds, but higher animals are more limited and rely in part on external nutritional sources. Mammals can convert some amino acids into other amino acids and also synthesize limited amounts in the gut, but must obtain most of the amino acids from food. Amino acids which must be derived from external food sources are termed *‘essential indispensable’*. If an essential amino acid is missing from the diet, growth, development and many other functions are impaired and eventually stop.

In humans, eight amino acids are essential and four are partially synthesized from other amino acids and are termed ‘*conditionally essential’.* Of the four amino acids implicated in the proposed hair-brain trade-off, *methionine* is essential and *cysteine, arginine* and *tyrosine* are ‘conditionally essential’. Protein requirements are measured in grams per kilogram body weight per day (g/kg/d) and called “recommended daily allowances” (RDA). Human adult maintenance needs are modest, at 56 g/d for a 70 kg person. Applied to a putative hominid, this would only represent 12-15% of the overall resting energy requirement. (FAO/WHO/UNU 2002; Food and Nutrition Board & Institute of Medicine 2002). Coincidentally these requirements are very similar to established Baboon requirements (15-20% according to the Merck Veterinary Manual).

However, demand greatly varies by age and activity. Infants, children and pregnant and lactating women require twice as much protein. Physical activity and climatic stress also increase needs several folds and the optimal amino acid requirements for maximal muscle growth are probably higher than those suggested for normal growth and physical activity (Lemon 1996; ADA Report 2000; Tarnopolsky 2004); see Table 1.

**Table 1:** **Human protein requirements (RDA)**

Age, years	Protein requirement	
	g/kg/d	Mean g/d
Neonates, 0 - 0.5^a^	1.52	5
Age 1-3^a^	1.1	16
Age 4-18^a^	0.95	40
Adults^a^	0.80	56
Pregnant and lactating^a^	1.55	110
Athletes^a^	1.2-1.8	90-130
Athletes^b^	3.1	220

^a^FAO/WHO/UNU ( 2002).

^b^Lemon ( 1996); ADA Report ( 2000); Burke ( 2001); Barr and Rideout ( 2004); Volek et al. ( 2006).

Protein requirements of a “Running Paleolithic Hunter”, can be assumed to be particularly high (Raichlen et al. 2011), and so would protein requirements during ontogenic growth spurts (Leigh 1996).

Since not all dietary protein contains all amino acids, quality is important. A steady and optimal supply of all essential amino acid in early childhood is vital for growth and behavior. An insufficient protein supply is the primary cause of cognitive and behavioral defects in malnourished infants; and, conversely, the increase in stature in affluent societies in the last century is largely attributed to enhanced protein availability (FAO/WHO/UNU 2002; Waterlow 1997; Meisel and Vega 2007). Amino acid requirements are particularly high in early infancy when most brain growth occurs and they fall substantially after maturation (Table 2). For example, arginine is essential only for infants and does not constitute a limiting factor after the brain reaches full size (Klein 2002; Pencharz and Ball 2004).

**Table 2:** **Estimated requirements of selected amino acids in humans by age**

	Requirement in mg/kg/d		
Amino acid^a^	0.5 y	11-14 y	Adult
Arginine^b^	70		
Histidine	22	12	10
Isoleucine	36	22	20
Leucine	73	44	39
Lysine	64	35	30
Methionine + cysteine^c^	31	17	22
Phenylalanine + tyrosine^d^	59	30	38
Threonine	34	18	23
Tryptophan	9.5	5	6
Valine	49	29	39

^a^FAO/WHO/UNU 2002, except for arginine (FAO/WHO/UNU 2002).

^b^Calculated according to minimal requirements of preterm infants (Klein 2002).

^c^Sulphuric amino acids (cysteine may be derived from methionine).

^d^When limited, tyrosine may be derived from phenylalanine.

### The amino acid shuttle

Ingested amino acids are metabolized, converted into body building proteins and then recycled over and over at very high rates in what is generally known as a “protein turnover” (Pfrimer et al. 2009). Ultimately, after many repeated shuttles, amino acids are oxidized and the nitrogenous residues are excreted as metabolic end-products in urine.

Beyond their role in body building, amino acids are vital for numerous metabolic processes that affect function and maintenance (Czikk et al. 2003; Wu 2009). It is therefore reasonable to assume that the ontogenetic encephalization process that occurs in infants requires much amino acid supplementation (Lepage et al. 1997; Buonocore et al. 2001; Reinstein and Ciechanover 2006) in addition to several other brain selective nutrients (Lynfield 1960). Furthermore, the speed of protein turnover largely determines its waste rates. The faster the shuttle, the higher is the rate of amino acid loss and the greater is the dependence on external nutritional sources. The brain thus accounts for much of the essential amino acid drain. This is important in large brained primates and vital in the much larger brained humans. In mammals, a substantial amount of cysteine and arginine are synthesized into keratin, a fibrous protein which is the main component of hair, nails, skin and horn.

Human hair is composed of about 17% cysteine, a sulphuric amino acid noted for its ability to add rigidity to biological tissue (Table 3). This transformation of amino acids into keratin is a one way street. Hair, nails and keratinized skin are dead structures. Proteins converted into them are lost to the body, much as protein in milk is lost in lactation. Therefore, the maintenance of a coat of hair constitutes a nutritional and metabolic drain of protein similar to the stress imposed on the female body by breast feeding. In fact, the added stress of lactation commonly produces a temporary hair loss in females (Lynfield 1960; Randall and Ebling 1991; Novak and Meyer 2009; Camacho-Martinez 2009).

**Table 3:** **Amino acid content of human hair protein performed by amino acid analysis of hydrolyzed proteins**

Amino acids^a^	Median and range within studies
	g/100 g hair
Cysteine + methionine + cysteic acid^b^	17 (8 –18.7)
Phenylalanine + tyrosine	4 (1.7 – 5.7)
Arginine	6.5 (2.5 – 9.3).

^a^See detailed data in the Table 7.

^b^In the mature hair cysteine is partially oxidized into cysteic acid.

### Metabolic considerations

Unlike most other tissue the brain functions steadily and largely independently of physical activity. Its effect on protein turnover primarily depends on its size. Over time, the brain requires more energy than equivalent muscle tissue (Leonard et al. 2003; Raichle and Mintun 2006). In most mammals the brain accounts for 0.5-1% of total body weight and the overall metabolic requirements do not exceed 5% of the “resting metabolic rate” (RMR). In human adults, the brain constitutes 2-3% of body weight and accounts for 20-25% of RMR. In infants, the brain can reach 10% of body weight and consume 60% or more of the energy expenditure; and the rate is even higher in fetus (Leonard et al. 2007; Barrickman and Lin 2010, Bogin 1997; Cunnane and Crawford, 2003). This high energy expenditure is associated with a greatly enhanced protein turnover and a dependence on a steady supply of essential amino acids (Sheffield-Moore et al. 2004; Bolster et al. 2005; Cuthbertson et al. 2006). The rate of protein turnover largely determines its waste rate. The faster protein cycles in body and brain, the greater is the amino acid loss and the consequent dependence on nutritional protein.

From a whole body perspective, the three organs with the greatest essential amino acid requirement are the brain, which depends on a steady amino acid flow, hair into which amino acids are lost and muscles. However, brain and hair have a constant need of amino acids while muscles require them intermittently during exertion. The interplay between the two constant structures, further orchestrated by protein intake, is meaningful in all larger brained species, is important in primates and is critical in humans where the energy requirements of a large brain dominate development.

The amino acids implicated in proposed hair-brain tradeoff are the sulphuric amino acids (methionine and cysteine), aromatic amino acids (tyrosine and phenyl alanine) and arginine [see Table 4]. These are involved in the brain in a wide set of vital functions which make their availability critical. The following list outlines their main functions to convey their critical roles:

**Table 4:** **Estimation of amino acid wearing away in hair by the adult pre-human hominid, average and (range)**

	Hair content^a^	Daily loss ^b^	Minimal requirement^c^
	%	g/d	g/d
Total hair wearing away		21 – 56	
Arginine	6.3 (6.1–9.3).	2.5 (0.5 – 9).	
Sulphuric amino acids^d^	17 (8-18.7).	6.5 (1.7 – 11).	1.5
Aromatic amino acids^e^	3.8 (2-7).	1.4 (0.4 – 3.4).	2.7

^a^See Table 7, median and range.

^b^According to Tables 2 and 5, median and range.

^c^Calculated according to Table 2 and assuming 70 kg body weight.

^d^Cysteine and methionine.

^e^Phenylalanine and tyrosine.

The sulphuric amino acids are mainly involved in methylation reactions such as:

- Methyl flux to facilitate myelin insulation and creatine synthesis (Bianchi et al. 1999; Pritzker et al. 2000; Cimato et al. 2002; Stead et al. 2006; Harauz and Musse 2007; Polverini et al. 2008; Harauz and Libich 2009).
- Mathyl flow for osmolite compound synthesis to enhance protein folding and antioxidant activities (Mcmanus et al. 1995; Hill et al. 2002; Lambert 2004).
- Methyl flux for myelin post translational modification to enhance conductance and electrical insulation. (Harauz and Libich 2009; Cao et al. 1999; Homchaudhuri et al. 2009).

The aromatic amino acids phenylalanine and tyrosine are recycled for:

- Tubulin tyrosination (Wadey et al. 2009; Heng et al. 2009; Janke and Kneussel 2010); the increase of signal conductance by tyrosine substitution of myelin basic protein; and the biogenesis of myelin membranes: sorting, trafficking and cell polarity (Baron and Hoekstra 2010).

Arginine is necessary for:

- Nitric oxide synthesis (NO) for physiological regulation of the nervous system and synaptic plasticity, learning and memory (Liu et al. 2009; Guix et al. 2005; Gensert and Ratan 2006).
- Citruline and agmatine synthesis and signaling molecule synthesis (Liu et al. 2009; Guix et al. 2005).
- myelin protein synthesis. (Harauz and Musse 2007; Polverini et al. 2008; Ridsdale et al. 1997; Hu et al. 2004).
- Arginine is also needed for creatine synthesis to help maintain brain energy homeostasis ( HMDB 3.0http://www.hmdb.ca/; Pfefferle et al. 2011).

In this context it is worth noting that proteins embedded in myelin and microtubles in the brain undergo intensive post-translational reactions. Such modified amino acids cannot be redirected into the protein turnover and are lost. The brain, therefore, needs a relatively higher amino acid supply than other organs.

Availability of essential amino acids thus constitutes a metabolic substrate barrier that is determined by ingested food on the one hand and by leaching into hair on the other. The maintenance of a full coat of hair is therefore in conflict with the requirements imposed by a large brain; and we hypothesize that brain growth in early hairy hominids would have been limited by the loss of these essential amino-acids into hair.

### Estimation of protein and amino acid supply for fur maintenance

To test this hypothesis we estimated what the amino acid loss would have been in a hypothetical large brained “hairy” hominid of modern human proportions. For convenience we chose a “standard” individual of 170 cm height and 70 kg weight (Auerbach and Ruff 2004; Ruff 2010). To enhance validity we calculated hair synthesis by two different convergent methods: by estimating hair production of single follicles and multiplying it for the whole body; and by estimating hair production of the scalp and projecting to the complete body. The two estimations yield close and partially overlapping estimates. We adopt the lowest and highest estimates as the most likely range for our calculations (Table 5).

**Table 5:** **Estimation of total body hair synthesis and amino acid wearing away by the pre-human hominid by two approaches**

A. Hair synthesis of a single follicle	
Body area (70 kg body weight; 170 cm height), cm^2^ ^a^	18000
Follicles density per cm^2^ ^b^	316
Total follicles	5.7 × 10^6^
Daily hair synthesis, μg per follicle^c^	5 – 10
Hypothetical whole body hair synthesis, g/d	28 – 56
**B. Total scalp hair production**	
Scalp hair follicles^d^	80 – 150 × 10^3^
Scalp hair production, g/d^e^	0.3
Total body follicles^f^	5.7 × 10^6^
Hypothetical whole body hair synthesis, g/d	21 – 40
Overall estimation, g/d	21 – 56

^a^Calculated for a 70 kg body weight and 170 cm height as follows, Body area (m^2^). = 0.00718 × kg^0.425^× height^0.725^ DuBois and DuBois ( 1915).

^b^Paus and Cotsarelis ( 1999); Sinclair et al. ( 2005); Krause and Foitzik ( 2006).

^c^Rogers ( 2004).

^d^Krause and Foitzik ( 2006).

^e^Galloway et al. ( 1971).

^f^Sinclair et al. ( 2005).

The estimation methods are as follows:

A. Estimation of protein loss from a single follicle:
  For a hair fiber of diameter 100 μm and a growth rate of about 20 μm per hour, 5-10 μg of protein are produced in a single follicle every 24/h (Rogers et al. 2004). With an assumed density of 316 follicles per cm^2^ [typical of the human head] and an assumption that the whole body is covered with the same density; a hair covered hominid of modern proportion would synthesize protein on a range of 28 to 56 g/d.
B. Estimation of protein loss from the complete scalp.

The number of follicles on the scalp is estimated at 80 – 150 × 10^3^, and the number of follicles on the body is estimated at 5.7 × 10^6^. Thus the ratio between the two is on a range of 1/71 to 1/133. Total hair production on the scalp is estimated at 0.3 g/d. Therefore we can estimate whole body protein synthesis of a hairy hominid on a range of 21 – 40 g/d.

Using these two estimates, we adopt a range of 21-56 g/d as a likely daily protein loss into hair. [A smaller large brained hominid would produce less protein but would expand a large proportion of the daily energy on brain function].

Using the estimated amino acid content in hair, presented in Table 3 and the protein loss into hair presented in Table 5 we can now calculate a daily loss of 6.5 grams of sulphuric amino acids, 2.5 grams of arginine and 1.4 grams of phenylalanine and tyrosine (Table 4).

The table also shows the daily nutritional requirements for sulphuric and aromatic amino acids (there are no nutritional guidelines argenine which is not an essential amino acid for adults). Some additional methionine would be further lost through the synthesis of methylated fatty acids in the hair (Breakspear et al. 2005); and humans also convert some sulphuric amino acids into taurine, a sulfonic acid which promotes hair growth and protects the brain against free radical damage (Collin et al. 2006); and some cysteine is diverted to glutathione production (Buonocore et al. 2001; Dringen 2000).

The daily loss of amino acids, in particular sulphuric amino acids, into hair (Table 4) constitutes a severe depletion of these amino acids and an overall protein loss which would be beyond the requirements of a large brained hairy hominid. The loss would be further compounded by physical activity (Lemon 1996; Tarnopolsky 2004) and seasonal requirements (Table 6). In fact, insufficient amino acid supply causes hair growth retardation even in modern humans (Elsas and Acosta 2014).

**Table 6:** **Amino acid content (sulfur amino acids, arginine and aromatic amino acids). of human hair genes with a high sulfur content (approximately g/100 g hair)**

	Keratin type	Sulphuric compounds^a^	Arginine %	Aromatic compounds^b^
MacKinnon et al. ( 1990).	High S	35.5%	2.4%	1.8%
Rogers et al. ( 2007).	KAP^c^	35.2%		
Emonet et al. ( 1997).	High S	32%	4.3%	
Yahagi et al. ( 2004).	KAP	33.4%		
Rogers et al. ( 2001).	KAP	30.7%		
Lee and Baden ( 1974).	Human p167	25.3%	6.3%	2.9%
Rogers et al. ( 2004).	KAP	25.1%		
Shimomura et al. ( 2002).	KAP	25%		
Median value		30.9%		

^a^See footnote 1 on Table 7.

^b^See footnote 2 on Table 7.

^c^Keratin associated proteins.

### Estimation of the amino acid composition of human hair

Amino acid composition in hair varies among individuals and over time. Therefore rates vary between published analyses. Several estimates of the “limiting” amino acids are listed in Table 7 with summary and median values.

**Table 7:** **Comparative composition of protein hair according to the data collected from 10 studies**

		Sulphuric compounds^a^	Arginine %	Aromatic compounds^b^
Caucasian, “brown hair”	Wolfram ( 2003).	18.2%	6%	3.6%
Asian	Wolfram ( 2003).	18.1%	6.1%	2.2%
African	Wolfram ( 2003).	16.8%	6.1%	4.2%
	Lang and Lucas ( 1952).	18.7%	8.9%	4.6%
	Masukawa et al. ( 2004).	17.6%	6.2%	3.6%
	Sass et al. ( 2004).	17.4%	6.7%	1.7%
	Bradbury ( 1979).	17.1%	6.3%	3.8%
	Nagase et al. ( 2008).	16.8%	5.9%	1.8%
	Jones et al. ( 1996).	16.5%	6.2%	3. 8%
	Zahn and Gattner ( 1997).	16.6%	6.5%	3.7%
	Crewther et al. ( 1965).	15.9%	6.2%	5.7%
	Kim et al. ( 2013)^c^.	14%		
	McCullagh et al. ( 2005).	11.3%	9.3%	7.3%
	Yu et al. ( 1993).	8%	7.2%	4.5%
	Akhtar et al. (1997).	8%	7.2%	4.5%
Median values		17%	6.5%	3.8%

^a^Methinine and cystein (methionine cannot derived from cysteine while cysteine might derived from methionine) and also cysteic acid which is a minor fraction derived from cysteine.

^b^Phenylalanine and tyrosine (phenylalanine cannot derived from tyrosine while tyrosine might derived from phenylalanine).

^c^According to the sulphur content of hair.

### The how and when of hair loss

Our simulation leads to a tentative conclusion that a dense coat of hair and a large brain cannot co-exist. The implication is that body hair must have been lost to allow the brain to grow. One alternative route would hold that hair was lost first and that brain grew later in an unrelated process. Alternatively an evolutionary drive for a larger brain may have begun before hair was lost and may have contributed towards the selection for less hair. In following hair loss it is important to note that humans have not lost the capacity to grow hair, still possess piliary appendages and produce substantial amounts of hair on body and head. The fact that hair follicles were retained and only hair growth was depressed, favors an interrelated selection in which less hairy individuals have an advantage over more hairy ones at a time of rapid brain growth. This explanation is attractive insofar as a fairly wide range of hair densities exists in many mammalian species making hair quantity and quality a ready selection variable (as evidenced by the easy artificial selection for hair and wool in sheep, goats, dogs, cats and other domestic animals).

The vital thermoregulatory functions of hair would require that hair could have been lost at a time and a place when insulation was of little importance. An equatorial African savannah is a natural candidate and has in fact been suggested by proponents of other theories on hair; i.e. the sweating and cooling theories (Amaral, 1996; Wheeler, 1996). The timing is unclear but may have coincided with a period of gradual warming. Since a large brain is subject to greater calcium ion stress and is better protected against it by the non hydroxilated form of sialic acid (Muthing et al. 1998; Martin and Freeze 2003) to which humans are disposed by a unique single mutation which occurred some 2.2-2.4 MYA (Varki 2007), one may perhaps see this point in time as the earliest boundary for initiation. Here too the assumed timing is attractive as it corresponds well with a host of major developments in human evolution including bipedalism (Isbell and Young 1996).

### The phylogeny and ontogeny of brain growth

The proposed evolutionary scenario raises several putative questions: Why have early humans not been driven to a greater protein consumption? Why have carnivore brains remained relatively small? Why don’t human adults develop body hair in maturity? These and similar questions can serve as guidelines to the evolutionary role of hair loss in humans and require a discourse on the broader evolutionary implications of the hair-brain duet. Encephalization comprises two main components, a phylogenic process by which brains in successive generations in a given population become progressively larger; and an ontogenetic process by which brains of developing individuals in any generation go through a period of intensive growth. The two are inter-connected. The further advanced the phylogenic process, the more intense must ontogenetic growth be to achieve a typical full brain size within the short pre-determined period of development.

We believe that the metabolic requirements of a large brain for sulphuric amino acids and arginine are in conflict with protein deposition and loss in hair. While this may not have hindered the initiation of a phylogenetic incremental brain development, process would have been slowed down by its ontogenetic complement. Assuming hairiness and baboon-like foraging, the available diet (Wheeler 1992; Washburn and McCown 1978) would not sustain fetal and neonate brain growth of human proportions. Natural selection might favor larger brains but dietary constraints would limit their expression (Babbitt et al. 2011; Wheeler 1992; Washburn and McCown 1978; Nagy et al. 1999).

Under such conditions, however, individuals with a less dense coat of hair might fare better in selective terms. We propose that if brain size had a survival value that was greater than the survival value conferred by a dense fur, the gene pool of individuals with larger brains would have increased at a greater rate than the gene pool of individuals with dense hair. Hairiness in the population would have been reduced while brain size increased thus in effect constituting a biochemical hair-brain trade-off.

The dietary constraints draw attention to dietary protein. Human diets are flexible and easily adapt to accommodate for climatic and environmental variables. It is reasonable to assume that omnivorous hominids faced with an amino acid crunch may have gravitated towards higher protein consumption. Indeed it is now estimated that animal protein constitutes some 30% of total energy intake in some extant chimp societies (Stanford 1996; Schoeninger et al. 1999; Gilby et al. 2006) and high protein requirements are listed for several other primates (Committee on Animal Nutrition 2003). Such protein consumption rates are similar to modern adult humans and should be sufficient to support both brain and hair. However, high rates of animal protein are difficult to maintain by active non-carnivores and it seems that neither total vegetarianism nor committed carnivory afford balanced nutritional intakes that allow species to depart from an entrenched nutritional tract. Both strategies are subject to periodic “food scarcity” bottlenecks, and each produce certain deficiencies and enhances specific risk factors. Nutritional specialists at both ends of the spectrum are least flexible and most vulnerable (Wheeler 1992; Speth 2010). Some tendency towards nutritional opportunism seems to enhance evolutionary plasticity and an omnivorous strategy may be the best overall solution. Using dietary nutrient analysis we may rule out brain growth in committed grazers, leaf eaters, frugivores and specialist terrestrial carnivores (the case is not clear for marine mammals). Potential candidates include most true omnivorous species including humans, chimps and other higher primates.

Of these, only humans lost body hair and even they may be viewed more as hair suppressors than out and out hair losers. The fact that humans only suppress hair growth leaving the follicle intact, supports our contention that the developmental conflict is limited to the production of hair and amino acid loss.

When viewed from a nutritional perspective, the extended human gestation may reflect, in part, a moderating evolutionary adaptation to the conflict between ontogeny and phylogeny. A longer pregnancy may slow the protein drain on the mother thereby lowering nutritional stress (Rosenberg 1992). This may also explain why modern humans have not revitalized hair growth in cold climates. Once hair growth was suppressed to ensure optimal fetal and infant brain development, the ability to reselect hair in adulthood may have become an evolutionary dead end in selective terms.

Selective hair loss gives rise to several tempting sociobiological scenarios. If hair loss and big brains go hand in hand it may be supposed that less hairy big brained individuals would be more likely to achieve high status and have greater reproductive success. This would further enhance the evolution of hairlessness and may speed it along within stable social groups. Furthermore, hair in modern humans has obvious social signal functions that go beyond mere cognition (Hopp 1983; Dunbar and Shultz 2007) it affects social recognition and helps shape attitudes towards conspicuity. Mate choice strategies in early humans may have led to a ‘naked is fitter’ preference and furthered a human predilection for hairlessness which in turn would promote the selection of less hairy individuals.

The proposed evolutionary scenario may tie together several hitherto unexplained variables and may also have some profound evolutionary implications. First it reinforces an intuitive hypothesis that encephalization could not have occurred in herbivores or in exclusively vegetarian primates. This may shed some light on the emergent findings on chimp predatory behavior. It has been proposed that chimps are protein deficient. We suggest that all larger brained primates must be sulphuric amino acid, aromatic amino acids and arginine deficient because of the brain-hair conflict. Second, the presumed trade-off would only be possible under warm and stable climatic conditions, where relative hairlessness would not constitute a survival disadvantage. This fits well with ubiquitous equatorial evolutionary concepts. It may also narrow the proposed timeframe of hair loss and may further resolve some of the debates on hair (Washburn and McCown 1978; Potts 1998; Hetherington and Reid 2010).

### Estimation of the amino acid composition of human hair

Amino acid composition in hair varies among individuals and over time. Therefore rates vary between published analyses. Several estimates of the “limiting” amino acids are listed in Table 7 with summary median summary values.
